# Diagnosis of infantile subglottic hemangioma: a 10-year experience of 25 cases

**DOI:** 10.3389/fped.2025.1499656

**Published:** 2025-05-30

**Authors:** Xiaoben Liang, Rong Xu, Hongming Xu, Jiarui Chen, Xiaoyan Li

**Affiliations:** ^1^ Department of Otolaryngology-Head and Neck Surgery, Shanghai Children's Hospital, School of Medicine, Shanghai Jiao Tong University, Shanghai, China; ^2^ Department of Radiology, Shanghai Children's Hospital, School of Medicine, Shanghai Jiao Tong University, Shanghai, China

**Keywords:** subglottic hemangioma, flexible fiberoptic laryngoscopy, contrast-enhanced computed tomography, infant, diagnosis

## Abstract

**Objectives:**

This study aims to explore the clinical appearances of infantile subglottic hemangioma (SGH) and the diagnostic value of flexible fiberoptic laryngoscopy (FFL) combined with contrast-enhanced CT (CECT).

**Methods:**

We retrospectively analyzed the data of 25 children diagnosed with SGH from January 2012 to January 2022.

**Results:**

FFL showed a smooth, rounded, vascular-appearing submucosal lesion in the subglottic wall, while CECT revealed an enhancing lesion, obscuring the airway lumen. Among the 25 cases (8 males and 17 females; 10 left-sided, 11 right-sided, and 4 middle), the clinical appearances contained stridor (25), respiratory distress (13), three-concave sign (10), barking cough (9), feeding difficulty (8), cyanosis (2), and hoarseness (2). SGH with cutaneous hemangiomas accounted for 24% (6/25). The age at presentation ranged from 1 day to 8 months (median, 33 days), including 96% (24/25) of cases aged <6 months. Moreover, 92% (23/25) of cases had a history of misdiagnosis, 22 respiratory infections, 5 laryngomalacia, 1 laryngeal cyst, and 1 asthma, individually or in combination. Except for one case that died of polygenic abnormality and another case lost to follow-up, the remaining 23 cases were cured after oral propranolol.

**Conclusions:**

For an infant with respiratory symptoms, who has repeated condition or poor effect after routine treatment, SGH should be considered, especially in infants under 6 months old. FFL combined with CECT is recommended to make a definite diagnosis of SGH.

## 1 Introduction

Subglottic hemangioma (SGH) is a rare form of infantile hemangioma and occupies barely 1.5% of all congenital laryngeal abnormalities (1). Despite its self-limiting natural course, SGH can damage the child's quality of life and even be life-threatening (2, 3). Clinical features are non-specific and mainly include recurrent stridor, respiratory distress, barking cough, repeated respiratory infections, cyanosis, thoracic, feeding difficulty, and abdominal retractions (4–7). Clinically, it is often misdiagnosed as a respiratory infection, but conventional anti-inflammatory treatment has poor effect or is repeated (8). Therefore, rapid and accurate diagnosis of SGH has extremely important significance (9, 10).

Clinically, SGH is prone to misdiagnosis before coming to our hospital, as many clinicians are not yet familiar with its clinical and imaging features. As a tertiary children's hospital, our Department of Otolaryngology—Head and Neck Surgery has certain advantages in treatment with SGH. Hence, we performed a retrospective analysis of the clinical data of SGH in the last 10 years to guide clinical practice.

## 2 Materials and methods

### 2.1 General information

In this study, we retrospectively analyzed 25 infants presenting with respiratory obstruction who were finally diagnosed with SGH by laryngoscopy combined with contrast-enhanced computed tomography (CECT) and treated with propranolol between January 2012 and January 2022. We collected data including clinical symptoms, gender, side, size, age at presentation, age at diagnosis, history of misdiagnosis, presence of other hemangiomas, diagnostic methods, treatment, and outcome. This study was approved by our institutional Research Ethics Board (2021R120-E01).

Inclusion criteria: (1) under 16 years old; (2) hemangioma located in subglottic alone or accompanied by other locations; (3) patients who underwent flexible fiberoptic laryngoscopy (FFL) and contrast-enhanced computed tomography (CECT); and (4) those who were treated with oral propranolol and followed up. Exclusion criteria: (1) patients with incomplete hospitalization and follow-up information; (2) repeated cases admitted to our hospital; (3) and those with other respiratory tract, thoracic, or pleural diseases.

### 2.2 Flexible fiberoptic laryngoscopy (FFL)

In our series, there were 22 cases initially misdiagnosed as respiratory infections (alone or combined with laryngomalacia, laryngeal cyst, or asthma), and they had poor effect or repeated condition after routine anti-inflammatory treatment. One case was misdiagnosed as merely laryngomalacia, and no improvement was found after treatment with calcium supplement and a small amount of multi-meal feeding. After otolaryngology consultation, the 23 infants underwent flexible fiberoptic laryngoscopy (FFL, Olympus Medical Systems, Tokyo, Japan). The remaining two cases were treated in the otolaryngology clinic and then underwent FFL.

### 2.3 Contrast-enhanced computed tomography (CECT)

The CT scan (GE LightSpeed VCT, USA) was performed on the neck, with a layer thickness of 0.625 mm, pitch of 0.984, interval of 2.5 mm, tube current of 240 mA, and tube voltage of 100 kV. Contrast enhancement was performed via iohexol administration, and CECT images were obtained in all 25 patients. Coronal and transverse multiplanar reconstruction CT images were reconstructed with a 3 mm section thickness. All cases were given 10% chloral hydrate (0.5 ml/kg) orally before examinations.

## 3 Results

### 3.1 Patient characteristics

**Figure 1:**
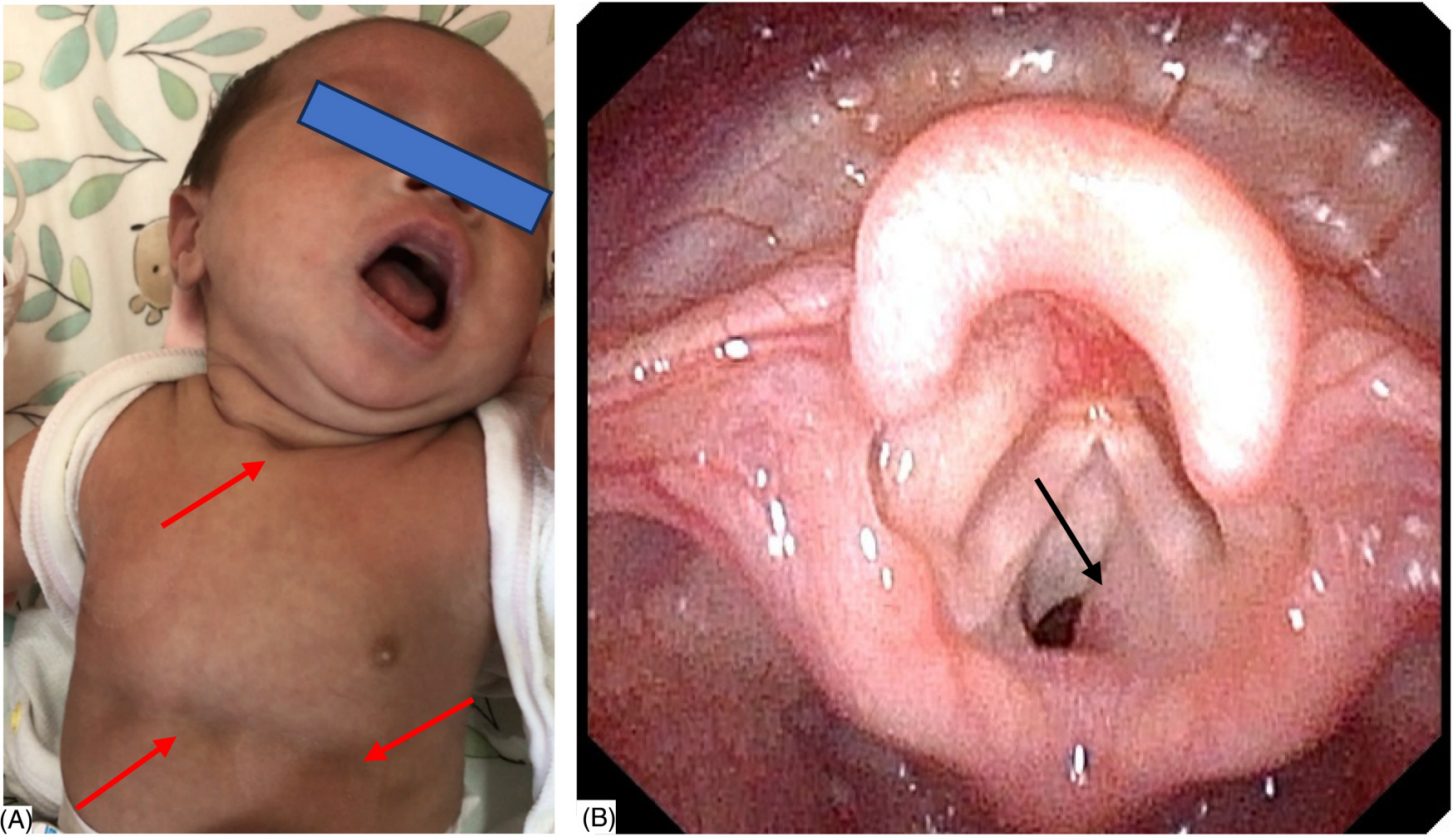
**(A)** A female of 40 days with stridor, respiratory distress, and three-concave sign (red arrow). **(B)** Flexible fiberoptic laryngoscopy (FFL) revealed a smooth, protruded mucosa with a pinkish hue lesion (black arrow) in the right lateral subglottic wall, obscuring the airway lumen.

Among the 25 cases, there were 17 females and 8 males. The age at presentation ranged from 1 day to 8 months, including 96% (24/25) of cases aged <6 months and 16% (4/25) of cases present at birth, with a median age of 33 days. The age at diagnosis ranged from 1 to 28.3 months, with a median of 3 months. The demographic details are listed in Table 1. There were 11 right-sided, 10 left-sided, and 4 middle SGH. Upper respiratory tract obstruction was the main clinical manifestation (Figure 1A), which included stridor (25/25), respiratory distress (13/25), three-concave sign (10/25), barking cough (9/25), feeding difficulty (8/25), cyanosis (2/25), and hoarseness (2/25), individually or in combination. The history of misdiagnosis was found in 23 cases, 22 respiratory infections (bronchitis/pneumonia/acute laryngitis), 5 laryngomalacia, 1 laryngeal cyst, and 1 asthma, alone or in combination. The cases of SGH combined with other multiple hemangiomas took up 24% (6/25), which were localized on the neck (1/25), back/buttocks (2/25), hands/ arms/ shoulder (3/25), and lips/tongue/eyelid (2/25).

**Table 1:** Patient data.

Patient	Gender	Side	Size (mm)	Plain CT (HU)	CECT (HU)	Age at presentation (days)	Clinical manifestations	Misdiagnosis	Age at diagnosis (days)	Treatment	Outcome	Location of other hemangiomas
1	M	Left	8.2	38	262	1	A	Pneumonia	90	Propranolol	Resolved	No
2	M	Left	5.3	78	288	33	A, D	Bronchitis	63	Propranolol	Resolved	No
3	F	Left	1.89	75	300	1	A, E	Bronchitis	37	Propranolol	Dead^a^	No
4	M	Right	3.8	50	230	1	A, B, C, F	Bronchitis	187	Intubation, propranolol	Extubation, resolved	No
5	M	Right	12	47	229	20	A	No	101	Propranolol	Resolved	No
6	F	Left	8.7	23	229	23	A, B, C, F, G	Pneumonia	53	Intubation, propranolol	Extubation, resolved	Lips/tongue
7	F	Right	5.8	68	255	79	A, B, C, E	Pneumonia	109	Propranolol	Resolved	Back
8	F	Right	4.5	45	205	58	A	Pneumonia	88	Propranolol	Resolved	No
9	F	Middle	6.5	52	190	36	A, D	Bronchitis, laryngomalacia	48	Propranolol	Resolved	No
10	F	Right	8	56	197	16	A, B, C, G	Bronchitis	39	Intubation, propranolol	Extubation, resolved	Hands
11	F	Middle	2.4	50	185	162	A, E	Laryngomalacia	252	Propranolol	Resolved	No
12	M	Right	10	51	118	240	A, D	Bronchitis, asthma	395	Propranolol	Resolved	No
13	F	Left	6.7	41	242	30	A, E	Bronchitis	71	Propranolol	Resolved	Upper arm
14	F	Right	5.8	47	195	28	A	Pneumonia	850	Intubation, tracheotomy^b^, propranolol	Decannulated, resolved	No
15	M	Left	7.4	52	262	60	A, B, C, D	Pneumonia	203	Propranolol	Resolved	Shoulder, back, arms, buttocks
16	F	Middle	8.7	47	185	20	A, B, D	Bronchitis	80	Propranolol	Resolved	No
17	F	Middle	11.7	28	211	105	A, B, D, E	Acute laryngitis	129	Propranolol	Lost	No
18	F	Right	6.5	39	130	30	A, B, C, E	Laryngomalacia, bronchitis	117	Intubation, propranolol	Extubation, resolved	Neck, tongue, eyelid
19	M	Left	7	45	240	62	A, B, C, D	Bronchitis	75	Intubation, propranolol	Extubation, resolved	No
20	F	Right	8	70	230	1	A, B, C, D	Bronchitis, laryngeal cyst	304	Propranolol	Resolved	No
21	F	Right	6.4	65	272	40	A, B, C	No	47	Propranolol	Resolved	No
22	F	Left	8.11	40	300	60	A, E	Pneumonia	183	Propranolol	Resolved	No
23	F	Left	3	32	280	20	A, B	Bronchitis	30	Propranolol	Resolved	No
24	M	Right	5.6	50	220	90	A, B, C	Acute laryngitis	115	Propranolol	Resolved	No
25	F	Left	7.2	45	230	36	A, D, F	Laryngomalacia, bronchitis	90	Propranolol	Resolved	No

A, stridor; B, respiratory distress; C, three-concave sign; D, barking cough; E, feeding difficulty; F, cyanosis; G, hoarseness.

^a^ Died of multiple genetic defects.

^b^ Before visiting our hospital, tracheotomy was performed in other hospitals.

### 3.2 Diagnostic methods

**Figure 2:**
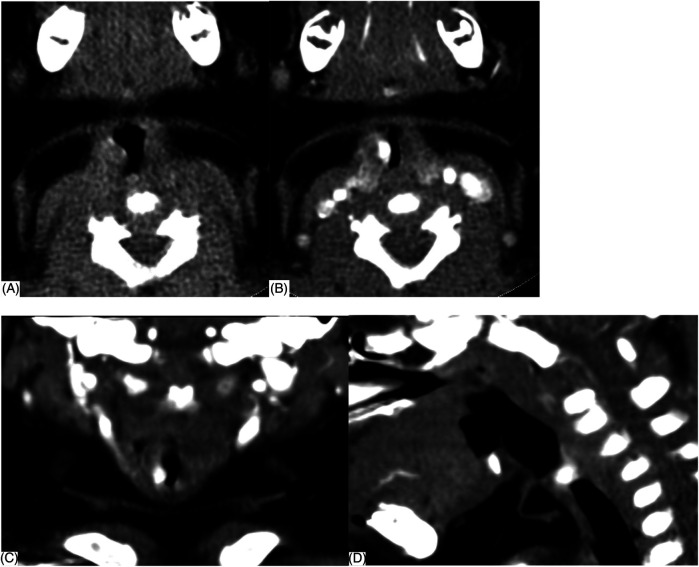
Contrast-enhanced CT (CECT) scan showing a well-defined enhancing lesion (arrow) in the submucosal location of the right lateral subglottic region. **(A)** Plain CT **(B)** transverse section, **(C)** coronal section, and **(D)** median sagittal section.

The final diagnosis of SGH was made by FFL and CECT. SGH was highly suspected if FFL showed a smooth, rounded, vascular-appearing submucosal lesion in the subglottic wall, obscuring the airway lumen (Figure 1B). The mucosa covering the SGH appeared pink (normal) in 21 (84%), a purple or bluish livid color in two (8%), and bright red in two (8%). CECT was performed subsequently, which showed an enhancing lesion in the submucosal location of the subglottic region, inducing significant airway obstruction likely SGH (Figure 2). The maximum diameter size of SGH ranged from 1.89 to 12 mm, with an average of (6.77 ± 2.53) mm. The mean plain CT value was (49.36 ± 13.66) HU, with a range of 23–78 HU, while the CECT value ranged from 118 to 300 HU, with an average of (227.40 ± 46.45) HU.

### 3.3 Treatment and follow-up

All 25 cases were subsequently treated with oral propranolol under adequate risk assessment after the diagnosis was confirmed without delay. The parents were advised to stop treatment when the case had no respiratory symptoms and the hemangioma disappeared by FFL (and/or) CECT. In this study, 24% (6/25) of patients presented with a history of intubation. Cases 4, 16, 18, and 19 underwent endotracheal intubation due to respiratory distress before diagnosis of SGH. Subsequently, extubation was performed successfully after taking propranolol for 3–7 days. In case 6, respiratory symptoms recurred on the 4th day after oral propranolol, endotracheal intubation was performed on the 7th day, and she was subsequently extubated on the 13th day. Case 14 underwent endotracheal intubation (42 days and 4 months, respectively), tracheotomy in other hospitals (5 months), oral propranolol (28 months), and then decannulated (30 months). Except for one case that died of polygenic abnormality and another case lost to follow-up, the remaining 23 cases were cured after oral propranolol.

## 4 Discussion

Although the SGH is a benign condition, it could be associated with a fatal outcome (2, 3). Clinically, the most common symptoms of SGH include biphasic stridor (8, 11, 12), followed by respiratory distress, barking cough (13), dysphagia (11, 13), thoracic and abdominal retractions, and hoarseness (5, 8). In our study, the symptoms included stridor (25/25), respiratory distress (13/25), three-concave sign (10/25), barking cough (9/25), feeding difficulty (8/25), cyanosis (2/25), and hoarseness (2/25), which were consistent with the above reports. Early clinical diagnosis of SGH is difficult, since the respiratory symptoms such as stridor may be misdiagnosed as respiratory infection, laryngomalacia, or asthma and may show improvement with anti-inflammatory therapy (8, 14). The symptoms of SGH may typically worsen in the presence of upper respiratory infection, therefore, mistaken for common disorders, such as infectious or inflammatory croup (8, 13). Chen (6) suggested we should consider SGH in infants under 2 years old presenting with respiratory symptoms, who had poor effect or repeated condition after anti-inflammatory treatment. In our series, 92% (23/25) had a history of misdiagnosis, 22 respiratory infections (bronchitis/pneumonia/acute laryngitis), 5 laryngomalacia, 1 laryngeal cyst, and 1 asthma, individually or in combination. Our research results were consistent with literature reports.

Laryngoscopy is a proven diagnostic tool for identifying airway narrowing, including at the subglottic level. Laryngoscopy can visualize the supraglottic and glottic regions to exclude causes of stridor such as severe laryngomalacia, glottic webs, and vocal cord palsy (15). Diagnosis is suggested by endoscopic observation of a unilateral smooth and soft lesion with a color that ranges from red to blue depending on the extent of the submucosal vascular proliferation (16). FFL demonstrates a classical mucosal blush and “bean-like” swelling in the subglottis (15), which is usually submucosal, asymmetric, and smooth (6). The endoscopic features of the tumor are a soft vascular-type swelling, the latter is mostly limited to the subglottis, between the mucus membrane and the perichondrium (2). FFL is less invasive and can be performed during awake respiration and has been proven to be an effective tool for visualizing the pediatric airway (10). In this study, FFL showed a smooth, rounded, vascular-appearing submucosal lesion in the subglottic wall, obscuring the airway lumen (Figure 1B). The mucosa covering the SGH appeared pink (normal) in 21 (84%), a purple or bluish livid color in 2 (8%), and bright red in 2 (8%). Our findings were consistent with the above reports.

**Figure 3:**
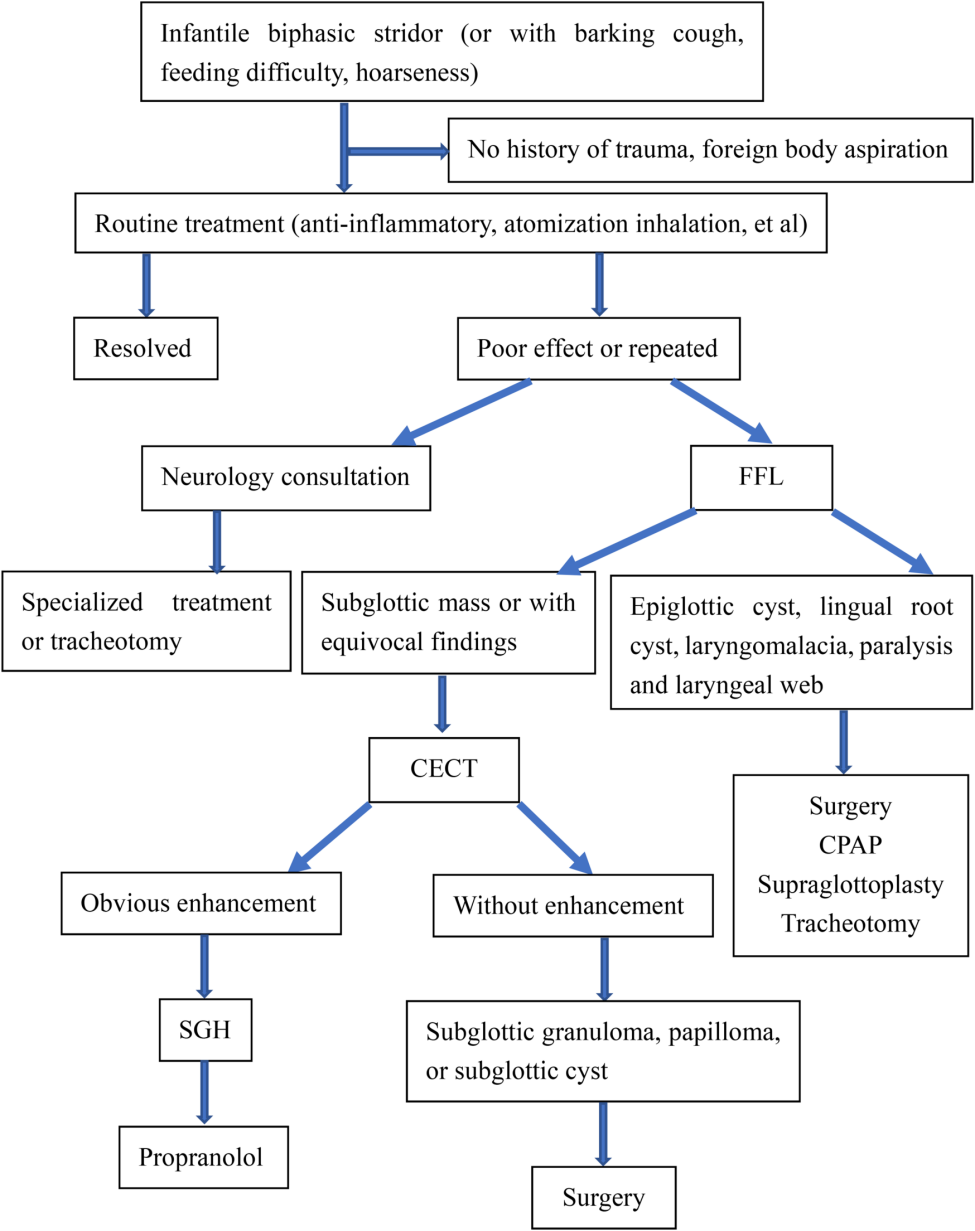
Diagnostic thinking in infantile SGH.

However, the endoscopic appearance is non-specific, and other primary tracheal tumors or highly vascularized metastatic tumors must be considered (17). The differentiated diagnosis of SGH in the infant should be expanded to include subglottic granuloma, papilloma, and subglottic cyst (6), and it is often difficult to differentiate by traditional endoscopic examination. Inflammatory change of the subglottic mucosa, as in spasmodic croup or severe gastroesophageal reflux, may mimic the endoscopic appearance of SGH. Therefore, complementary diagnostic techniques may provide benefits compared with laryngoscopy alone (14). When a child presents with stridor, laryngoscopy should be performed, followed by CT to rule out any type of vascular ring or mass (18). The diagnosis is confirmed by upper airway endoscopy (19) as well as the radiological examination and often obviates the need for biopsy (2). Imaging by CECT is helpful to appreciate the vascular nature of the lesion and determine the depth of invasion and extension of tracheal involvement (9, 11). Imaging can play a significant adjunct role in the evaluation of subglottic narrowing in an infant and exclude any external causes of compression (9, 15). CECT reveals SGH as a subglottic mass with intense, rapid enhancement as the cause of airway narrowing. The advantages of CECT are easy availability, multiplanar reconstructions, and non-invasive (8). In our study, CECT of the neck showed an isolated, well-defined enhancing lesion in the submucosal location of the subglottic region causing significant airway compromise (Figure 2). Here, we propose the diagnostic thinking, and advocate FFL combined with CECT to make a definitive diagnosis of SGH Figure 3. However, due to radiation risks and the need for sedation, CECT also has several drawbacks. Laryngeal US seems to be a rapid, tolerable, and highly reliable method worth further investigating (9).

The natural history of these tumors includes a stage of rapid proliferation during a 6- to 12-month period, followed by a stage of slow involution spreading over the following 18 months (20). Most SGH become symptomatic and could be life-threatening during their proliferative period. They grow quickly within the first few months following birth and lead to progressive airway obstruction (11, 21). Moreover, 80%–90% of affected babies present within the first 6 months of life, with a mean age of 3.6 months at diagnosis (22). The mean and median ages at diagnosis were 2.56 months and 2.0 months, respectively, with a range of 0.7–9 months (23). In our study, the age at presentation ranged from 1 day to 8 months, with a median age of 33 days. In addition, 96% (24/25) of cases presented <6 months old and 16% (4/25) of cases at birth. The median ages at diagnosis were 3 months, with a range of 1–28.3 months. Our research results were consistent with the above literature.

SGH may occur independently or accompany other cutaneous hemangiomas (19). Skin hemangiomas are present in 50% of cases of SGH at the time of diagnosis, with the head and neck being the most common location (23). In our study, the cases combined with other multiple hemangiomas accounted for 24% (6/25), which were localized on the neck (1/25), back/ buttocks (2/25), hands/arms/shoulder (3/25), and lips/tongue/eyelid (2/25). For reasons unknown, the incidence of SGH is lower in males than in females (24). There is a female preponderance (4, 13, 25). Schwartz et al. (21) found that 73% (36/49) of SGH cases published between 2009 and 2016 were female patients. Our series had 17 females and 8 males, which was inconsistent with the above reports.

Since the advent of propranolol therapy in 2008, the treatment of SGH has undergone a dramatic transformation (26). In 2009, Jephson et al. (27) reported the first two cases of SGH successfully treated with propranolol. Propranolol has since become the first-line treatment for SGH (5, 23, 28, 29) and has been shown to reduce the need for tracheostomy in these children (8, 30). In this study, except for one case that died of polygenic abnormality and another case lost to follow-up, the remaining 23 cases were cured after oral propranolol. The intubated patients received oral therapy through nasogastric tubes. Our results supported propranolol as the first-line therapeutic modality for SGH.

## 5 Conclusion

We advocate a strong index of suspicion for SGH presenting with respiratory symptoms, especially infants under 6 months old who have repeated condition or poor effect after routine treatment. We recommend FFL combined with CECT to make a definite diagnosis of SGH.

## Data Availability

The raw data supporting the conclusions of this article will be made available by the authors, without undue reservation.
